# Interprétation délicate de l'immunofixation des protéines sériques

**DOI:** 10.11604/pamj.2018.30.130.13662

**Published:** 2018-06-14

**Authors:** Raoul Karfo, Elie Kabré, Nadia Safir, Mounya Bouabdellah, Laila Benchekroun, Jean Sakandé, Layachi Chabraoui

**Affiliations:** 1 Service du Laboratoire Central de Biochimie, Centre Hospitalier Universitaire Ibn Sina, Maroc; 2 Unité de Formation et de Recherche en Sciences de la Santé, Université Ouaga I Pr Joseph Ki-Zerbo, Burkina Faso; 3 Clinique du Laboratoire de Biologie, Centre Médical du Camp General Aboubacar Sangoulé Lamizana, Ouagadougou, Burkina Faso; 4 Laboratoire de Biochimie, Centre Hospitalier Universitaire Yalgado Ouedraogo, Ouagadougou, Burkina Faso; 5 UFR de Chimie Biochimie et Biologie Moléculaire, Faculté de Médecine et de Pharmacie, Université Mohammed V, Rabat, Maroc

**Keywords:** Immunoglobuline monoclonale, électrophorèse, immunofixation, cryoglobuline, gammapathie monoclonale, Monoclonal immunoglobulin, electrophoresis, immunofixation, cryoglobuline, monoclonal gammopathy

## Abstract

L'immunofixation est actuellement très utilisée dans les laboratoires d'analyses médicales. L'interprétation des résultats est en général facile, mais il existe des cas qui posent des problèmes d'interprétation. Nous vous rapportons deux observations d'interprétation délicate. Dans la première observation, nous présentons un cas de précipitation non spécifique de la protéine sur toutes les pistes, dans la deuxième observation un cas présentant une double bande monoclonale à l'immunofixation. Pour ces deux observations, l'utilisation de la solution réductrice de β2-mercaptoéthanol nous a permis de résoudre le problème et de conclure à un diagnostic. La confrontation des données cliniques, radiologiques et biologiques est nécessaire avant de conclure à une immunoglobuline monoclonale.

## Abstract (en)

Immunofixation is currently very used in medical laboratories. The interpretation of the results is usually easy, but some cases raise interpretative problems. We here report two cases difficult to interpret. In the first case, we report a case of nonspecific precipitation of the protein on each track, in the second case we report a case of double monoclonal band on immunofixation electrophoresis. Reducing agent such as β2-mercaptoethanol used in these two cases allowed to solve the problem and to make a diagnosis. A comparison between clinical radiological and laboratory test data is necessary before making a diagnosis of monoclonal immunoglobulin.

## Introduction

L'immunofixation des protéines est une technique immunochimique connue depuis 1969 [1], après le principe de l'électrophorèse des protéines, connu depuis les premiers travaux de Tiselius dans les années 1930 [2]. C'est une technique qualitative appliquée à la révélation et l'identification des immunoglobulines monoclonales dans le sérum, les urines et éventuellement le liquide céphalorachidien. L'homogénéité structurale des molécules constituant l'immunoglobuline monoclonale implique une homogénéité de charge électrique, d'où une mobilité électrophorétique étroite propre à chaque immunoglobuline monoclonale. Cette caractéristique biochimique est à la base des différentes techniques actuellement utilisées pour distinguer l'immunoglobuline monoclonale des immunoglobulines polyclonales normales. La découverte d'une immunoglobuline monoclonale n'est pas synonyme de malignité certaine. On retrouve des cas de production d'immunoglobulines monoclonales en dehors de toute maladie maligne avérée (infections virales, bactériennes ou parasitaires, pathologies inflammatoires,…). C'est la conjugaison de plusieurs critères cliniques, radiologiques, hématologiques, biochimiques et immunochimiques qui permettent d'affirmer le caractère malin [3]. Une immunoglobuline monoclonale est une immunoglobuline produite en quantité anormale par un clone unique de lymphocytes B. Elle est constituée soit d'une seule classe de chaîne légère et de chaîne lourde (immunoglobuline entière), soit de chaînes légères isolées d'un seul type ou plus rarement de chaînes lourdes (en général fragmentaires) d'un seul type [4]. Les modalités du diagnostic immunochimique ont été précisées dans une revue de Lapalus et Chevailler [5]. Elles reposent sur la réalisation d'une analyse conjointe du sérum et des urines par au moins une électrophorèse (recherche et affirmation d'une homogénéité de charge), une immunofixation (identification de son isotypie), auxquelles on adjoindra le dosage pondéral des immunoglobulines pour estimer l'hypogammaglobulinémie résiduelle. Cette technique, en raison de sa simplicité et sa sensibilité, est actuellement de plus en plus utilisée dans les laboratoires d'analyses biomédicales. Avec la connaissance dont nous disposons aujourd'hui sur les protéines, et le développement des performances techniques, l'interprétation des résultats est en général facile, mais il existe des cas qui posent des problèmes d'interprétation. A travers notre pratique quotidienne au laboratoire central de biochimie du centre hospitalier universitaire (CHU) Ibn Sina, de Rabat-Salé (Maroc), nous allons présenter dans un but didactique et pédagogique deux cas d'interprétation délicate.

## Méthodes

L'immunofixation (IF) est réalisée à la suite d'une découverte de pic effilé à base étroite à l'électrophorèse migrant dans la zone des γ, β, ou α2 globulines; d'une hypogammaglobulinémie; ou devant des renseignements cliniques évoquant une gammapathie monoclonale chez un patient en cas d'une électrophorèse des protides normale. L'analyse se fait sur des échantillons frais. Afin d'éviter des phénomènes de zone par excès d'antigènes, les échantillons de sérum sont utilisés dilués. Le système d'immunofixation sérique qui a été utilisé est le système Sebia* constititué du coffret Hydragel IF* et de l'automate Hydrasis*. Le coffret est composé de mèches tamponnées, de l'hydragel, de l'applicateur, du colorant acide violet, du diluant pour l'échantillon du patient, de trois types de papier filtre, et d'une solution de lavage. Son principe est le suivant séparation des protéines par électrophorèse, précipitation des différentes immunoglobulines à l'aide d'anticorps monospécifiques (G, A, M, K, L) et enfin coloration des immunoglobulines précipitées. Le gel est composé de six pistes différentes. La première piste sert de référence grâce à la précipitation de toutes les protéines (ELP). Les cinq autres pistes permettent de caractériser la ou les bandes monoclonales grâce à des anticorps spécifiques anti-chaînes lourdes (G, A, M) et anti-chaînes légères kappa (K) et lambda (L). Selon les recommandations du fabricant, la dilution du sérum se fait avec le diluant fourni dans le coffret (bleu de bromophénol). La dilution est variable en fonction des pistes: dilution au 1/6 pour la piste IgG car ce sont les immunoglobulines majoritaires dans le sérum et dilution au 1/3 pour les autres pistes. Les dilutions sont adaptées en cas d'hypergammaglobulinémie ou d'hypogammaglobulinémie. Le volume d'antisérum appliqué est de 40 μL pour la piste ELP (électrophorèse des protéines totales) et 25 μL pour A, G, M, K, L. L'immunofixation permet la visualisation des différents clones d'immunoglobulines (IgG, IgA, IgM et chaînes légères kappa et lambda) du sérum. Lorsqu'à l'immunofixation des protéines sériques, on met en évidence une bande monoclonale sur la piste des chaînes légères kappa ou lambda sans correspondance sur la piste des chaînes lourdes G, A, M, on procède à la recherche des chaînes légères libres monoclonales sériques. En cas de négativité de la recherche de la protéine de Bence-Jones, une nouvelle immunofixation des protéines sériques est réalisée à la recherche d'immunoglobuline monoclonale de type D et de type E. Le sérum est dilué au 1/3, le volume d'antisérum appliqué est de 40 μL pour la piste ELP (électrophorèse des protéines totales) et 25 μL pour les pistes D, E. Certaines protéines et chaînes légères tendent à se polymériser et à former des agrégats. Dans ce cas, on traite l'échantillon (sérum, urine) avec une solution réductrice de β2-mercaptoéthanol (BME): pour le sérum, préparer la solution réductrice en mélangeant le BME avec le fluidil*, ajouter ensuite 25 μl de la solution réductrice préparée à 75 μl de sérum pur, agiter au vortex et laisser en contact 15 minutes minimum (30 minutes maximum), puis faire une immunofixation comme décrit précédemment.

## Résultats

### Cas 1: Polymérisation des protéines au niveau de la zone de dépôt

Madame BZ âgée de 60 ans, de sexe féminin et hospitalisée en médecine interne pour une thrombophlébite. L'électrophorèse des protéines sériques réalisée révèle la présence d'un pic effilé à base étroite migrant dans la zone des gammaglobulines. Une immunofixation des protéines sériques est faite en vue de typer l'immunoglobuline monoclonale et montre une polymérisation des protéines au point de dépôt au niveau de toutes les pistes (ELP, G, A, M, K, L) ce qui a nécessité un traitement préalable du sérum par le BME. Après avoir réalisé une deuxième immunofixation sur le sérum traité par la solution réductrice, on a pu typer l'immunoglobuline monoclonale qui était une IgG kappa.

### Cas 2: Gammapathie biclonale ou immunoglobine monoclonale à deux niveaux de polymérisation

Monsieur BG, de sexe masculin, âgé de 67 ans est reçu dans la salle de prélèvement de l'hôpital à titre externe pour suspicion de myélome multiple. L'électrophorèse des protides sériques a révélé la présence d'un pic effilé à base étroite migrant dans la zone des béta globulines. Une immunofixation des protéines sériques a été réalisé en vue de typer l'immunoglobuline monoclonale et a montré la présence d'une double bande monoclonale sur la piste des IgA et sur la piste des Kappas. Après traitement réducteur du sérum par le BME, une nouvelle immunofixation a été réalisée. Elle a montré la disparition de la deuxième bande monoclonale, ce qui nous a permis de porter le diagnostic d'une immunoglobuline monoclonale de type IgA Kappa à deux niveaux de polymérisation.

## Discussion

Le premier cas, soulève le problème de précipitation des protéines au point de dépôt ce qui rend une immunofixation pratiquement ininterprétable. Ce problème peut s'expliquer d'une part par la présence d'additifs dans le gel d'agarose: le polyéthylène glycol, composé qui vise à optimiser la réaction antigène-anticorps peut être la cause d'interférence en précipitant certaines protéines présentes dans le sérum et en empêchant les immunoglobulines de migrer librement dans le gel. L'utilisation de coffrets ne contenant pas ce type d'additifs peut pallier à ce genre de problème [6,7]. Certains auteurs proposent l'utilisation des antisérums d'une autre firme commerciale en sachant que la qualité des antisérums est susceptible de variations inter-lots. Il est donc judicieux pour un laboratoire réalisant des analyses d'électrophorèse et d'immunofixation de disposer d'antisérums de deux firmes différentes à utiliser en fonction des besoins. Par ailleurs, l'utilisation de fluidil et de BME s'avère utile [7]. Dans notre cas, nous avons résolu le problème par le traitement préalable des échantillons par le BME en association avec le fluidil*. L'utilisation du BME lors de la suspicion de la présence d'immunoglobulines monoclonales polymérisées permet de couper les ponts disulfures et d'obtenir une molécule monomérique et finalement d'homogénéiser sa mobilité électrophorétique. Le BME est adapté pour réduire les ponts disulfures des protéines. Le clivage intermoléculaire (en sous-unités) des ponts disulfures permet aux sous-unités d'une protéine de se séparer de façon indépendante. Le clivage des ponts disulfures intramoléculaires (au sein de la sous-unité) permet à la sous-unité de devenir complètement dénaturée. D'autre part, les bandes de précipitation au niveau de toutes les pistes d'un gel d'immunofixation peuvent évoquer la présence de cryoglobulines, d'immunoglobulines de classe IgM, ou de complexes IgM-IgG précipitant au dépôt [8]. La présence d'une cryoglobuline va donner une bande identique au niveau de chacune des pistes car les protéines restent immobilisées dans le gel et ne peuvent pas migrer normalement. Dans ce cas, le sérum devra être placé à 37°C avant d'être analysé dans des conditions techniques particulières. Les difficultés d'interprétation de l'immunofixation en raison de la polymérisation des protéines se rencontrent plus souvent avec l'IgM, molécule pentamérique de haut poids moléculaire mais peuvent même se voir avec les chaînes légères des immunoglobulines. Le deuxième cas, soulève la problématique de la conduite à tenir face à la présence de deux bandes monoclonales sur la plaque de l'immunofixation. L'utilisation du BME est proposée et est même déterminante [7]. La solution réductrice permet d'une part de confirmer le profil obtenu avec l'immunofixation si les bandes monoclonales persistent, après traitement du prélèvement par le BME. D'autre part le traitement réducteur permet d'infirmer et de corriger la première immunofixation comme c'est cas de notre observation où on a noté la persistance d'un seul pic après traitement du sérum par le BME, ce qui nous a permis de conclure à la présence d'une immunoglobuline monoclonale de type Ig A Kappa sous une forme monomère et sous une forme polymérisée [9].

## Conclusion

L'immunofixation est une technique dont l'interprétation est facile et simple, cependant des difficultés liées à des paramètres techniques ou au prélèvement existent. Il est plus que nécessaire pour les laboratoires qui utilise cette technique en routine de mettre en place des protocoles standards et simples pour éviter la répétition des tests coûteux, et qui retardent la transmission des résultats aux cliniciens. Les immunofixations présentant des difficultés d'interprétation doivent bénéficier d'une attention particulière de la part du biologiste afin d'éviter les pièges diagnostiques. Par ailleurs, nous rappelons la nécessité de la confrontation des données cliniques et radiologiques avec les résultats biologiques avant de conclure à la présence d'une immunoglobuline monoclonale. Enfin, nous suggérons également l'assistance et l'accompagnement des jeunes biologistes par des biologistes expérimentés lors de l'interprétation des résultats.

### Etat des connaissances actuelles sur le sujet

- Au Burkina Faso, la fréquence des myélomes multiple est croissante;
- Pour le biologiste, la difficulté essentielle consiste à affirmer l'origine monoclonale d'une immunoglobine détectée en grande quantité dans le sérum. L'affirmation du caractère monoclonal repose sur l'observation du pic à l'électrophorèse: aspect étroit et haut et Les examens complémentaires: l'immunofixation des protéines. Les difficultés d'interprétations connues sont celles de toutes les réactions antigènes-anticorps, la nature du sérum et l'importance du contexte clinique et biologique.

### Contribution de notre étude à la connaissance

- Ce travail fournit des informations précieuses aux biologistes pour la réalisation et l'interprétation de l'immunofixation surtout en ce qui concerne la nature du sérum.

## Conflits d'intérêts

Les auteurs ne déclarent aucun conflit d'intérêts.
